# Supplementary material for: Analysis and study of risk factors related to the progression of non-alcoholic fatty liver disease: A retrospective cohort study
Source: PLoS One. 2025 May 7;20(5):e0322990. doi: 10.1371/journal.pone.0322990 (PMC12057958; doi:10.1371/journal.pone.0322990)
Supplement: Attachment — Submitted filename: Response to Reviewers.docx [file pone.0322990.s002.docx]

**Response to Reviewers**

Dear editors and reviewers,

We sincerely appreciate your constructive comments and suggestions on our manuscript, titled "Analysis and Study of Risk Factors Related to the Progression of Non-Alcoholic Fatty Liver Disease: A Retrospective Cohort Study" (ID: PONE-D-24-34142). Your feedback has been invaluable in improving the quality and clarity of our manuscript.

In the revised manuscript, we have made significant updates based on your insights. Below, we provide detailed responses to each comment. We hope these revisions meet your expectations and address all concern

The main revisions in the new manuscript are:

1. We ensured consistency in the use of abbreviations, resolving discrepancies such as "FBS" vs. "FBG."

2. Recent literature has been integrated, and an extended discussion of potential mechanisms underlying the observed associations has been included.

3. For sample size calculation and efficacy analysis, we have provided the sample size calculation formula to illustrate the reasonableness of our sample size, and I will clarify the specific formula in my reply to reviewer 1.

4. Contradictions in the discussion section, particularly about the relationship between age, visceral fat area (VFA), and NAFLD progression in the elderly population, have been resolved after reviewing relevant references.

5. The manuscript was reviewed and edited by native English-speaking experts to improve language clarity and accuracy.

Sincerely,

ZhenHua Zhou and Co-Authors

**Response to the academic editor**

Academic Editor Comments to the Author (Required): The statistical methodology needs more rigorous documentation, particularly regarding sample size calculation and power analysis. The Results section would benefit from a more consistent presentation of statistical findings, with standardized reporting of significance levels and effect sizes. Some contradictions appear in the Discussion section, particularly regarding the relationship between age, visceral fat area (VFA), and NAFLD progression in elderly populations, which requires clarification. The manuscript would be substantially strengthened by a more comprehensive integration of recent literature, particularly in comparing findings with similar studies. The discussion of potential mechanisms underlying the observed associations could be expanded to provide better context for the findings. Some technical issues need addressing, including standardization of abbreviations (e.g., FBS vs. FBG) and professional English language editing to improve clarity and precision.

Response from the Authors:

Thank you for your valuable suggestions. The manuscript has been carefully and thoroughly revised in response to the reviewers’ comments. All the questions raised by the reviewers have been addressed.

In the revised manuscript, the abbreviations for FBS and FBG have been clarified and used consistently throughout. Additionally, we have updated several references to include more recent studies, such as metabolic risk factors and incident advanced liver disease in non-alcoholic fatty liver disease (NAFLD): A systematic review and meta-analysis of population-based observational studies published in PLOS Medicine. We sincerely apologize for the inconsistencies in the discussion section of the original manuscript, particularly regarding the relationship between age, visceral fat area (VFA), and NAFLD progression in the elderly population. After reviewing relevant references, these inconsistencies have been removed, and the discussion section has been revised to ensure clarity and logical coherence. We have also incorporated recent literature to provide a more comprehensive analysis and have expanded on the potential mechanisms underlying the observed associations. Furthermore, we engaged a professional English editor to revise the entire text, which has resulted in the correction of numerous grammatical errors and improvements to the overall readability.

**Response to the Reviewer # 1**

Dear reviewer, thank you very much for your interest in our findings and for pointing out the flaws in the analyses. We have addressed your concerns in a point-by-point manner below, and hope that you will find the added information suitable and sufficient for publication.

1. The result section in abstract is not clear. Please revise this section.

**Response from the authors:**

Thank you for pointing out these problems. We apologize for the lack of clarity in the description of the results in the abstract. We revised the Results section of the abstract to ensure clarity and conciseness.

2. How sample size was calculated?

**Response from the authors:**

Thank you for your insightful question. The sample size was calculated using the formula for infinite populations in cross-sectional epidemiological research. For a reference prevalence rate of 40% (Shanghai population), a permissible error of 0.05, and an initial sample size of 576, we included 545 participants after accounting for follow-up losses. The specific formula has been detailed in the Methods section. The formula is shown below：

3. A language editing is recommended by a native speaker to revise some grammatical errors.

**Response from the authors:**

Native English-speaking experts revised the manuscript to correct grammatical errors and improve language quality.

4. Please update some previous references with new references.

**Response from the authors:**

The authors agree with the reviewer. We thank the reviewer for the highly valuable comment. Recent references, including new studies from 2020–2024, were added to enhance the manuscript's relevance and context.

**Response to the Reviewer # 2**

Dear reviewer, thank you very much for your interest in our findings and for pointing out the flaws in the analyses. We have addressed your concerns in a point-by-point manner below, and hope that you will find the added information suitable and sufficient for publication.

1. On page 13, Part 3 (Results), Section 3.1 (Comparison of general data and biochemical indicators of the three groups), the first paragraph stating "There were statistically significant differences…….. (Table 1; P < 0.05)" requires a fundamental revision. Additionally, the placement of abbreviated words needs correction, such as using 'FBS' for blood lipids, which is incorrect, and using 'TC' as an abbreviation for blood lipids (Do you mean Total Cholesterol? and GGt is Gamma-Glutamyl Transferase

**Response from the authors:**

Thank you for your insightful question. We have changed the wording of Part 3 (Results), Section 3.1. This is the latest statement in the paragraph: the differences in age (AGE), alanine aminotransferase (ALT), aspartate aminotransferase (AST), alkaline phosphatase (ALP), gamma-glutamyltransferase (GGT), triglycerides (TG), fasting blood glucose (FBG), and visceral fat area (VFA) among the three groups of patients who were changed to the new group were statistically significant (P < 0.05). However, the differences in gender, total cholesterol (TC), low-density lipoprotein (LDL) and high-density lipoprotein (HDL) were not statistically significant (Table 1).

2. In the Results section, it's recommended to place the significance level (P < 0.05) at the end of sentences discussing significant results, rather than following those addressing non-significant findings.
**Response from the authors:**

p-values (e.g., p < 0.05) are now placed at the end of sentences discussing significant results, per your suggestion.

3. In table 1, please add the Age unit in the table.

**Response from the authors:**

Thank you for pointing out these problems. Age units (years) have been added to Table 1 for clarity.

4. There is an inconsistency regarding ALP in the explanation. According to Table 5, with a p-value of 0.034, ALP is significant at the conventional level of 0.05. However, it is incorrectly stated as not significant for individuals over 60 years. This needs correction to reflect the data accurately.

**Response from the authors:**

We thank the reviewer for the highly valuable comment. We are very sorry, this is a writing error on our part. ALP in Table 5, with a p-value of 0.034, is significant at the conventional level of 0.05. The authors have revised in the literature that ALP is statistically significant in individuals over 60 years of age.

5. Please use consistent abbreviations throughout the text for a specific topic. For instance, you have used FBS throughout the article, but FBG once at the beginning of the discussion. Using different terms confuses the readers.

**Response from the authors:**

Thank you for pointing out these problems. "FBG" is now used consistently throughout the manuscript instead of "FBS."

6. These two parts in the article's discussion section about elderly people appear to be contradictory. Please review them carefully and make corrections as needed:
“This is entirely consistent with a previous study in which the cumulative incidence of NAFLD increased with VFA in both men and women, and further analyses showed that an increase in VFA especially in those younger than 60 years of age led to an increase in NAFLD incidence. In contrast, these differences disappeared in those over 60 years of age.”
And,
“In the elderly population, age is an independent risk factor for NAFLD disease progression, which is consistent with some studies in recent years. This may be because aging is accompanied by abdominal obesity and excess visceral fat, which leads to an increase in insulin resistance and pro-inflammatory cytokine secretion, and the accumulation of excess fat in the liver and the molecular mechanisms of hepatocellular damage caused by aging include increased ROS formation, DNA damage, which aggravates NAFLD.”
**Response from the authors:**

Thank you for pointing out these problems. We have corrected the contradictory parts of the article regarding the discussion of older adults by removing the parts that contradicted the above after reviewing the relevant references, as corrected in the discussion section of the article. Correct the article from “In the elderly population, age is an independent risk factor for NAFLD disease progression, which is consistent with some studies in recent years. This may be because aging is accompanied by abdominal obesity and excess visceral fat, which leads to an increase in insulin resistance and pro-inflammatory cytokine secretion, and the accumulation of excess fat in the liver and the molecular mechanisms of hepatocellular damage caused by aging include increased ROS formation, DNA damage, which aggravates NAFLD” to “This is because the molecular mechanisms by which aging leads to hepatocellular damage include increased ROS formation and DNA damage, which aggravates NAFLD”

7.A more effective comparison with similar studies could be incorporated into the discussion section of the article, potentially drawing on the findings from other relevant research. For example, the results of the following article can be helpful in the discussion section:
“Metabolic risk factors and incident advanced liver disease in non-alcoholic fatty liver disease (NAFLD): A systematic review and meta-analysis of population-based observational studies,2020”

**Response from the authors:**

The authors agree with the reviewer. Thank you for your kind suggestion. We have added “Metabolic risk factors and incident advanced liver disease in non-alcoholic fatty liver disease (NAFLD): A systematic review and meta-analysis of population-based observational studies” to my article , which is serial number 17 in the reference. Your suggestions are very helpful.

8. There are few typographical or grammatical errors that should be corrected. Please, check.
**Response from the authors:**

The authors agree with the reviewer. Thank you for your kind suggestion. We invited native English-speaking experts in the field of liver disease to touch up this article to improve clarity and accuracy. Thank you for these suggestions to make our articles more valuable!

**Response to the Reviewer # 3**

We feel great thanks for your professional review work on our article.

1. The data and results of the article"Analysis and study of risk factors related to the progression of non-alcoholic fatty liver disease" are not valuable for publishing in this journal, considering the population, indicators, duration of data collection, and the journal's standards.

**Response from the authors:**

Thank you for your valuable feedback on our manuscript titled "Analysis and study of risk factors related to the progression of non-alcoholic fatty liver disease." We deeply appreciate the time and effort you have taken to evaluate our work and would like to address your concerns in detail.

Population and Data Characteristics:

Our study analyzed clinical data from NAFLD patients over one year in a real-world setting. While we understand the concern regarding the population size and data collection duration, the selected cohort represents a snapshot of patients from a high-volume hospital specializing in integrative medicine. The study focused on the clinical indicators commonly used in NAFLD diagnosis and management, and we believe these findings provide valuable insights into the risk factors for disease progression. These results may contribute to understanding region- and population-specific patterns, which could be further validated in larger, multicenter studies.

Clinical Indicators:

The indicators used in our analysis (e.g., ALT, TG, FBG, and VFA) are widely recognized in clinical practice and research as significant markers for NAFLD progression. By employing stratified analyses by gender and age, we provided a nuanced exploration of their effects across different subgroups, which could have practical implications for personalized prevention and treatment strategies.

Duration of Data Collection:

The one-year data collection period was chosen to ensure data reliability and reflect routine clinical practice within the studied population. While we acknowledge that a longer follow-up could provide additional insights into disease progression, the retrospective cohort design allowed us to identify independent risk factors within this timeframe. These findings may serve as an important foundation for prospective studies with extended observation periods.

Alignment with Journal Standards:

We carefully reviewed the journal's scope and standards before submission, and we believe that our study aligns with its focus on advancing knowledge related to liver disease. The identification of independent risk factors for NAFLD progression, along with sex- and age-specific insights, adds value to the current literature. However, we remain open to revising the manuscript further to better meet the journal's expectations.

In summary, while we recognize the limitations of our study, we believe that the data and results provide meaningful contributions to the understanding of NAFLD progression. We are willing to address any specific concerns raised by the journal or reviewers and welcome suggestions for further improving the manuscript.

**We hope these detailed responses and revisions address all your concerns. Thank you for your time and effort in reviewing our manuscript.**

Sincerely,

ZhenHua Zhou and Co-Authors
