# Supplementary material for: Analysis and study of risk factors related to the progression of non-alcoholic fatty liver disease: A retrospective cohort study
Source: PLoS One. 2025 May 7;20(5):e0322990. doi: 10.1371/journal.pone.0322990 (PMC12057958; doi:10.1371/journal.pone.0322990)
Supplement: Attachment — Submitted filename: renamed_70fe3.docx [file pone.0322990.s003.docx]

**Response to Reviewers**

Dear editors and reviewers,

We sincerely appreciate your constructive comments and suggestions on our manuscript, titled "Analysis and Study of Risk Factors Related to the Progression of Non-Alcoholic Fatty Liver Disease: A Retrospective Cohort Study" (ID: PONE-D-24-34142). Your feedback has been invaluable in improving the quality and clarity of our manuscript.

In the revised manuscript, we have made significant updates based on your insights. Below, we provide detailed responses to each comment. We hope these revisions meet your expectations and address all concern

The main revisions in the new manuscript are:

1. we have revised lines 6 and 7 of the introduction according to the actual content of the original reference 4 (now reference 5), and corrected our misunderstanding of reference 4 (now reference 5).

2. in section 3.1 “Comparison of General Data and Biochemical Indicators of the Three Groups”, the confusion caused by the inconsistency was corrected by stating that the total number of patients was 330, the number of young and middle-aged patients was 204, and the number of elderly patients was 126.

3. in the second paragraph of the Discussion section we followed the reviewer's suggestion to first address the explanations related to ALT, then FBS, then TG, and finally VFA. this resolves a serious confusion in the reporting and analysis of the findings in the Discussion section.

4. we cited some recent references to support our article. references 3, 7, 8, and 31 are the most recent references that we cited.

Sincerely,

ZhenHua Zhou and Co-Authors

**Response to the academic editor**

Academic Editor Comments to the Author (Required): Please address reviewers' comments.

Response from the Authors:

Thank you for your valuable suggestions. The manuscript has been carefully and thoroughly revised in response to the reviewers’ comments. All the questions raised by the reviewers have been addressed.

We have revised lines 6 and 7 of the introduction to the actual content of the original reference 4 (now reference 5) to correct our misunderstanding of reference 4 (now reference 5). We then corrected the confusion caused by the inconsistency in Section 3.1, “Comparison of general data and biochemical indicators among the three groups”, by stating that the total number of patients was 330, the number of young and middle-aged patients was 204, and the number of elderly patients was 126. We have further addressed the serious confusion in the reporting and analysis of the findings in the Discussion section. We cite a number of recent references to support our article.

**Response to the Reviewer # 1**

Dear Reviewer, Thank you very much for your time and valuable feedback on our manuscript titled "Analysis and Study of Risk Factors Related to the Progression of Non-Alcoholic Fatty Liver Disease: A Retrospective Cohort Study." We sincerely appreciate your constructive comments, which have helped us improve the quality and clarity of our work.

We are grateful for your insightful questions and thorough review, which have significantly strengthened our study. Your expertise has been invaluable in shaping this manuscript into a more rigorous and polished piece of research.

Once again, thank you for your support and for contributing to the improvement of our work.

**Response to the Reviewer # 2**

Dear reviewer, thank you very much for your interest in our findings and for pointing out the flaws in the analyses. We have addressed your concerns in a point-by-point manner below, and hope that you will find the added information suitable and sufficient for publication.

1. Introduction, lines 6 and 7: "The prevalence is also increasing yearly with improving people's quality of life" This sentence does not appear to be correct. Upon reviewing the referenced article, this concept was not mentioned. Please provide the relevant paragraph from reference 4, where this concept was derived, including the page number, in the reviewer response for verification. Otherwise, this sentence must be revised based on the actual content of reference 4.

**Response from the authors:** Thank you for pointing out this problem. Due to our carelessness, we misinterpreted the meaning of reference 4, and we have now corrected our error in citing reference 4 (now reference 5) by revising lines 6 and 7 of the introduction to follow the actual content of the original reference 4 (now reference 5).
2. In section 3.1, 'Comparison of the three groups of general data and biochemical indicators,' there is inconsistency that cause confusion and need to be clarified. At the beginning of the text, the total number of patients is stated as 330. Then, in the age classification section, the number of young and middle-aged patients is stated as 204, and the number of elderly patients is stated as 126, which sums up to 330. However, the text separately states that 204 young patients and 126 elderly patients were examined. This type of separation and presentation can be misleading.

**Response from the authors:** We fully agree with your suggestion. We have corrected the confusion caused by an inconsistency in Section 3.1, “Comparison of General Data and Biochemical Indicators of the Three Groups,” by stating that the total number of patients was 330, the number of young and middle-aged patients was 204, and the number of elderly patients was 126. We hope that you will accept our revision.
3. The correct procedure for writing the discussion section of a scientific report is to analyze the findings based on the order in which they are mentioned. Consequently, it is preferable from the second paragraph of the discussion to first address the explanations related to ALT, followed by FBS, then TG, and finally VFA, rather than initially focusing on weight. The reporting and analysis of findings within the discussion section suffer from significant disorganization. Please revise and rearrange them according to the order specified above.

**Response from the authors:** Thank you for pointing this out. In the second paragraph of the Discussion section we first address the explanations related to ALT, then FBS, then TG, and finally VFA. we have now resolved the serious confusion in the reporting and analysis of findings in the Discussion section.

**Response to the Reviewer # 3**

We feel great thanks for your professional review work on our article.

1. In my opinion, after the revisions, this study does not meet the scope and size requirements of this journal based on its sample size and methodology.

**Response from the authors:**

Thank you for your valuable feedback on our manuscript titled "Analysis and study of risk factors related to the progression of non-alcoholic fatty liver disease." We deeply appreciate the time and effort you have taken to evaluate our work and would like to address your concerns in detail.

About the study design and data characteristics:

This study used a retrospective cohort design to systematically analyze clinical data from NAFLD patients over one year. Although the study period was relatively limited, we ensured the reliability and representativeness of the data through strict inclusion criteria and quality control measures. The results of the study not only reflect the real situation in clinical practice but also provide an important reference for subsequent larger prospective studies. Our sample size was calculated using the formula for an infinite population in cross-sectional epidemiologic studies. For a reference prevalence rate of 40% (Shanghai population), a permissible error of 0.05, and an initial sample size of 576, we included 545 participants after accounting for follow-up losses. The specific formula has been detailed in the Methods section. The formula is shown below：

In terms of indicator selection:

We focused on clinically used indicators such as ALT, TG, FBG, and VFA, which are markers that have been confirmed to be closely associated with NAFLD progression by several studies. Through stratified analysis methods, we further revealed the specific performance of these indicators in different populations, providing a more targeted reference for clinical practice.

Research Value and Outlook:

We fully recognize the limitations of this study in terms of sample size and follow-up time. However, it should be emphasized that these preliminary findings provide new clues for in-depth exploration of the pathogenesis and progression pattern of NAFLD. We plan to expand the sample size and extend the observation time in the follow-up study to further validate the current findings.

We fully agree with your journal's strict requirements for research quality and have completely revised the paper according to the review comments. We believe that the revised paper is more in line with the publication standards of your journal and can provide valuable references for researchers in related fields.

We hope these detailed responses and revisions address all your concerns. Thank you for your time and effort in reviewing our manuscript.

Sincerely,

ZhenHua Zhou and Co-Authors
